# Positional In-Match Running Demands of University Rugby Players in South Africa

**DOI:** 10.3389/fpsyg.2020.01591

**Published:** 2020-07-16

**Authors:** Cameron Donkin, Ranel Venter, Derik Coetzee, Wilbur Kraak

**Affiliations:** ^1^Department of Sport Science, Stellenbosch University, Stellenbosch, South Africa; ^2^Department of Sport and Exercise Sciences, University of the Free State, Bloemfontein, South Africa

**Keywords:** GPS, rugby union, positional groups, student athletes, performance analysis

## Abstract

The implementation of the Varsity Cup rugby competition among South African universities has sparked interest in university rugby cultures around the country. The Varsity Cup has established itself as one of the most important rugby competitions for full-time students. The development of specific conditioning programs for rugby players requires a thorough understanding of the game and the unique demands of playing positions. Therefore, the study aimed to investigate the in-match running demands of South African university rugby players by using GPS during match play for primary and secondary positional groups. Rugby players (*n* = 40) from two universities were assessed during match play (*n* = 17) over a competitive season by using GPS. Players were grouped into two primary positional groups, forwards (*n* = 22) and backs (*n* = 18), and five secondary positional groups, tight forwards (*n* = 14), loose forwards (*n* = 8), half backs (*n* = 5), inside backs (*n* = 6), and outside backs (*n* = 7). The GPS analysis provided the following match-play movements: total distance, high-speed meters, maximum velocity, match intensity, the number of accelerations and decelerations, and velocity zone. Halfbacks recorded the highest total distance (6620.9 ± 784.4 m; *p* = 0.02) and match intensity (77.7 ± 11.6 m/min; *p* = 0.01). Outside backs recorded the highest maximum velocity (8.385 ± 1.242 m/s; *p* = 0.00). Loose forwards registered the highest number of accelerations (385.5 ± 122.1) and decelerations (378.7 ± 108.1). Backs and their specific subgroups play at and within high velocity thresholds, significantly higher (*p* = 0.01) than that of the forwards. Forwards tend to be involved in a higher amount of accelerations and decelerations during match play, suggesting that forward play is at close quarters to the opposition. During university rugby matches, the backs covered greater distances and speeds than the forwards, whereas the forwards achieved more accelerations and decelerations than backs. Results from the study can assist Varsity Cup strength and conditioning coaches to prepare players for the position-specific demands of the competition.

## Introduction

Rugby union (“rugby”), as an intermittent, high-intensity, collision sport that is popular globally, is characterized by physically intense phases of play and displays of speed, skill, and strength (Lindsay et al., 2015). In South Africa, rugby is enjoyed by players of all ages (Harris and Wise, 2011). Universities in South Africa have become part of an inter-university competition (Varsity Cup) that allows students to compete against one another on a semi-professional level. The Varsity Cup (VC) rugby competition in South Africa has become the most innovative and stimulating competitions in South Africa in terms of ideas (rules and law changes) and match play. The VC has expanded since it commenced in 2008 and has developed into a high-intensity, physically high-demanding competition at a university level. Furthermore, all the players are full-time students and have a full academic program. Nine university teams partake in a round-robin format to determine the top four university teams that would progress to knockout rounds. Teams play an equal number of matches both home and away over the 9-week round-robin format with one bye before the knockout stages of the competition. Limited information on the management of players to perform on all the demands of being a student athlete in South Africa has been published. However, since the onset of professionalism in rugby in 1995, the demands of the game have increased. The game became more intense, with players becoming heavier, and the backs particularly became taller (Quarrie and Hopkins, 2007; Vahed et al., 2014). The number of impacts and impacts at high speed (McLellan et al., 2011; Owen et al., 2015) and increased ball-in-play time (Schoeman and Coetzee, 2014) contributed to the changing demands of the sport. These increases could predominantly be attributed to changes in laws and improved match analysis, equipment technology, and player conditioning (Quarrie and Hopkins, 2007).

Along with increased demands on players, the demands differ per position played (Duthie et al., 2003; Austin et al., 2011; Cahill et al., 2013; Tee and Coopoo, 2015; Tee et al., 2017). Players are generally separated into two primary positional groups, namely, backs and forwards, and further subdivided into secondary positional groups discussed later in the “Materials and Methods” section (Duthie et al., 2003). All players are required to perform core actions/activities, such as tackling and rucking, during a match (Duthie et al., 2003), but the technical and tactical demands on players are different for the playing positions (Owen et al., 2015; Tee et al., 2018). Technically, backs focus on handling, passing, kicking, and running, whereas forwards focus on body position in contact and ball carrying. Tactically backs may aim to beat the defense using skillful moves and speed, where forwards may look to physically beat opponents to gain field advantage. To determine these position-specific characteristics, it is often difficult due to the dynamic movement of opposition players; accurate methods to quantify in-match running demands are required. Rugby as a sport takes place in a complex dynamic setting (Rothwell et al., 2017). In such a system, recorded player data for specific metrics become dependent on the actions of the opposition, known as the ecological and dynamic systems approach (Vilar et al., 2012). Although not a focus of this specific study, the understanding of the dynamic systems approach can assist in the understanding of recorded metrics as well as specific skill acquisition (Vilar et al., 2012; Rothwell et al., 2017). To develop specific conditioning programs and recovery strategies for rugby players, a thorough understanding of the game and the unique demands of different playing positions is essential (Venter et al., 2011).

A modern method used to quantify these positional demands is through global positioning systems (GPS). Research on players’ primary positional demands (Cunniffe et al., 2009) revealed that the total distances covered by elite rugby players per game by forwards were 6,680 m, and by backs, 7,227 m. Other studies confirmed that forwards accumulated 5,850 m (Cahill et al., 2013), 6680 m (Cunniffe et al., 2009), and 5,370 m (Cunningham et al., 2016) of total running distance. The backs accumulated total running distances of 6,545 m (Cahill et al., 2013), 7,227 m (Cunniffe et al., 2009), and 6,230 m (Cunningham et al., 2016). Austin et al. (2011) reported that the total distances covered by secondary positional groups during the Super Rugby tournament were as follows: front row (4,662 ± 659 m); back row (5,262 ± 131 m); inside backs (6,095 ± 213 m); and outside backs (4,774 ± 1,017 m). Yamamoto et al. (2017) found in a study on Japanese professional rugby players that the distance covered for the front row was 5604.0 m, second and third row forwards 5690.2 m, scrumhalf’s 7001.0 m, and backs 6072.3 m. However, little research has been carried out that quantified the running demands of university rugby players per positional group. Particular attention should be paid to the specific physical requirements of players in different positions to ensure that they receive adequate training (Tee et al., 2017) and individualized recovery.

GPS uses wearable microtechnology to record geographic positions over time and create a movement profile for players (Owen et al., 2015). Using this form of player movement analysis, individual external player loads can indicate specific positional demands of these players. The purpose of any rugby-specific physical training program should be to optimally prepare players for the demands of match play (Owen et al., 2015; Tee et al., 2018). This can be achieved by maximizing training specificity through the manipulation of training activities to simulate or exceed the skill and physical demands of the match (Tee et al., 2018). Tee et al. (2018) noted that physical preparation should not be done in isolation but with all the subsequent components for performance. Training sessions should be based around a combination of physical and mental aspects of the game that emulate match specificity, such as moving at high velocities and making decisions based on stimuli, such as defenders (Tee et al., 2018). The purpose of the current study was to give strength and conditioning coaches an indication of the match-specific positional running demands of South African university rugby players to develop and execute specific training sessions to maximize performance. Performance profiling of players participating in university rugby is necessary to establish normative values; the performance profiles can assist strength and conditioning coaches in monitoring players’ readiness for competition. Additionally, teams with the means to monitor training sessions can maximize performance and minimize injury risk through analyzing player load. Repeated exposure to high-speed running, accelerations, and decelerations will increase both injury risk and player load. Therefore, the current study aimed to investigate the in-match running demands of South African university rugby players by using GPS during match play for primary and secondary positional groups.

## Materials and Methods

### Participants

A total of 40 male rugby players from two South African universities competing in the 2018 VC rugby competition volunteered to participate in the study (Table 1). A total of 17 matches were played by the two teams against different teams and each other, both home and away for the competition duration. All players were informed about the purpose of the study and gave informed consent before participating in the study. The participants’ demographic information is shown in Table 1. Data were excluded from the study if a participant played less than 60 min or the GPS device lost signal. A total of 271 observations were included based on the inclusion and exclusion criteria.

**TABLE 1:** Demographic information of participants per positional group.

Positional group	n	Age (years) M ± SD	Height (cm) M ± SD	Weight (kg) M ± SD	Observations n
Combined	40	20.77 ± 0.44	179.55 ± 1.21	91.52 ± 0.83	271
Forwards	22	20.75 ± 0.61	183.34 ± 1.04^	101.24 ± 1.13^	147
Backs	18	20.78 ± 0.06	177.02 ± 0.75	85.05 ± 0.52	124
Tight forwards	14	20.84 ± 1.36	183.10 ± 7.7^cde^	107.05 ± 8.83^cde^	94
Loose forwards	8	20.65 ± 2.58	183.58 ± 5.63^cde^	95.43 ± 6.57^cde^	53
Half-backs	5	20.72 ± 1.48	175.22 ± 5.87	81.10 ± 7.68	36
Inside backs	6	20.67 ± 1.62	178.33 ± 4.05	88.43 ± 7.43	36
Outside backs	7	20.95 ± 1.57	177.50 ± 5.01	85.60 ± 8.64	52

**^ denotes significant differences in weight between primary positional groups. ^*a*^ denotes significant differences when comparing tight forwards to other secondary positional groups. ^*b*^ denotes significant differences when comparing loose forwards to other secondary positional groups. ^*c*^ denotes significant differences when comparing half backs to other secondary positional groups. ^*d*^ denotes significant differences when comparing inside backs to other secondary positional groups. ^*e*^ denotes significant differences when comparing outside backs to other secondary positional groups (p* ≤ *0.05).**

Players were categorized according to their playing positions and divided into primary positional groups described by Cahill et al. (2013) and Owen et al. (2015), namely, forwards (loose-head prop, hooker, tight-head prop, locks, blind-side flanker, open-side flanker, and eighth man) and backs (scrum-half, fly half, left winger, inside center, outside center, right winger, and fullback). The grouping was assumed to accurately reflect similar match demands for the playing positions and allow comparison with previous research. The secondary positional grouping subdivided the team into five groups, similar to the study by Owen et al. (2015). The five secondary positional groups for the current study consist of the tight forwards (loose head prop, hooker, tight-head prop, and locks), loose forwards (blind-side flanker, open-side flanker, and eighth-man), half backs (scrumhalf and fly half), inside backs (inside and outside centers), and outside backs (right- and left-wingers and fullbacks). The current study grouped the scrum and fly halves into one group called the half backs. The two positions were combined because the studies by Quarrie et al. (2013) and Tee and Coopoo (2015) reported no significant differences between the two playing positions. Table 2 presents the in-match running demands variables used in the current study. Only three velocity zones were measured as these zones were seen to best describe the running demands of rugby players, where velocity zones 1 and 2 represented low-velocity movements such as walking. The Health Sciences Research Ethics Committee (HSREC) (UFS-HSD2017/0062) at the University of the Free State approved the study.

**TABLE 2:** In-match running demands variables used in the study.

Variables	Description
Total distance (m)	Total distance describes the total number of meters covered by a player during the match. The metric is measured by the displacement of the GPS unit over time.
High-speed meters (m)	High-speed meters refer to distance covered at high speed. For the purpose of the current study, all meters ran above 5.56 m/s were registered as high-speed meters. Similar to total distance, the time between displacements of different samples when recording data indicates the type of meterage registered.
Maximum velocity (m/s)	Represents the maximum velocity achieved by a player during the match. Maximum velocity is registered at the peak of the player’s running performance.
Match intensity (m/min)	Measured as meters covered per minute, this metric represents an internal calculation of the GPS software that determines the meters ran over the duration of the match and equates it as match intensity. Cummins et al. (2013) similarly referred to relative distance (with reference to match intensity) as a calculated workload based on the overall distance covered per minute.
Number of accelerations (n) and decelerations (n)	Accelerations and decelerations indicate a change in speed over the established velocity zones. Accelerations are registered because of increases in speed through the velocity zones and decelerations are recorded because of decreases in speed through the velocity zones.
Velocity zones	Velocity zones 3 (4.44–5.56 m/s), 4 (5.57–6.94 m/s), and 5 (>6.95 m/s) were indicative of the total distance covered within the velocity zones.

### Data Collection Procedure

Players from the two universities were fitted with Catapult Minimax X4 10 Hz GPS units for the matches (Catapult; Melbourne, Australia). These two universities made use of the Catapult Minimax X4 10 Hz GPS units with the same velocity zones allowing for comparisons to be made on players’ performance. It should also be noted that the use of GPS units during matches is not common within the VC setting. The GPS units fitted into specialized neoprene vests designed specifically for positioning and securing of the unit on the player’s upper back. The GPS unit was switched on before the start of the warm-up and switched off after the match. The warm-up and half-time data were excluded and discarded. The data were analyzed to identify information regarding player demands as presented in Table 3 during match play in primary and secondary positional groups. Data were extracted and divided into the relative periods from the GPS units, using the Catapult Open Field (version 1.21.1) software (Catapult; Melbourne, Australia). Data were extracted as a csv file for further clean-up in a Microsoft Excel spreadsheet before being analyzed by the Statistica 13 data processing package (version 13.0.159.8) (Dell Inc., Round Rock, TX, United States).

**TABLE 3:** Positional demands for different variables per match during the 2018 Varsity Cup tournament (mean ± standard deviation).

Positional groups	Total distance (m)	High-speed meters (m)	Maximum velocity (m/s)	Match intensity (m/min)	Accelerations (n)	Decelerations (n)	VZ^3^ (4.45–5.56 m/s)	VZ^4^ (5.57–6.94 m/s)	VZ^5^ (>6.95 m/s)
Forwards	5734.4 ± 693.7	158.1 ± 30.8	6.598 ± 0.944	68.6 ± 9.5	363.8 ± 132.2	358.5 ± 117.7	197.4 ± 48.0	65.15 ± 24.5	27.9 ± 12.7
Backs	6261.6 ± 745.2	459.2 ± 58.7^	7.865 ± 1.001^	72.9 ± 11.4	367.9 ± 114.3	361.7 ± 96.4	305.3 ± 65.4^	158.3 ± 40.6^	71.5 ± 30.8^
Tight Forwards	5352.9 ± 798.6	69.7 ± 22.2	6.066 ± 1.079	66.8 ± 10.4	342.2 ± 142.3	338.4 ± 127.3	259.9 ± 43.7	61.2 ± 18.2	8.5 ± 7.8
Loose Forwards	6115.9 ± 588.8	246.4 ± 39.4	7.129 ± 0.809^a^	70.4 ± 8.5	385.5 ± 122.1	378.7 ± 108.1	529.5 ± 52.2^a^	199.2 ± 30.6^a^	47.2 ± 17.5^a^
Half Backs	6620.9 ± 784.4^a^	349.3 ± 54.5^a^	7.279 ± 0.866^a^	77.7 ± 11.6^ab^	366.0 ± 115.5	362.8 ± 98.8	760.6 ± 88.7^abcd^	291.3 ± 46.4^a^	58.0 ± 18.6^a^
Inside Backs	6084.6 ± 779.4	471.9 ± 55.8^abc^	7.930 ± 0.895^abc^	71.2 ± 11.3	363.3 ± 116.2	356.5 ± 98.6	573.9 ± 57.5^a^	321.1 ± 37.6^a^	150.9 ± 31.9^ab^
Outside Backs	6079.5 ± 671.7	556.5 ± 65.8^abc^	8.385 ± 1.242^abc^	69.7 ± 11.4	374.5 ± 111.2	365.9 ± 91.8	497.0 ± 49.8^a^	336.6 ± 37.8^ab^	219.9 ± 41.9^abc^

*,^^a, b, c, d, e^ indicates significant differences. ^ indicates differences for primary positional groups. ^a, b, c, d, e^ indicates differences for secondary positional groups.*

### Data Analysis

The Statistica 13 software package was used to process the data. Participant information was described by using descriptive statistics (mean [M] ± standard deviation [SD]). Mixed model ANOVA was used with “player” and “player^∗^period” as random effects, and “position,” “period,” and “position^∗^period” as fixed effects. Fisher least significant difference (LSD) testing was used for *post-hoc* testing. Normality assumptions were evaluated by inspecting normal probability plots and were mostly found to be acceptable. Participants were grouped according to primary and secondary positional groups. The position^∗^period effect was found to be not significant in all cases, so the results for the position effect are reported.

## Results

Results for all recorded metrics and per positional group are presented in Table 3. When comparing the primary positional groups, backs recorded significantly higher totals for high speed meters (*p* = 0.01), maximum velocity (*p* = 0.01), and velocity zones 3 (*p* = 0.01), 4 (*p* = 0.01), and 5 (*p* = 0.01). No significant differences were observed for the total distance covered and the number of accelerations and decelerations.

When comparing secondary positional groups, significant differences were observed for all metrics except for the number of accelerations and decelerations between the different subgroups. Half backs covered the highest total distance, significantly more (*p* = 0.02) than tight forwards. The outside backs covered the most high-speed meters (556.450 m), significantly more than all secondary positional groups (*p* = 0.01). The outside backs recorded the highest maximum velocity (8.385 ± 1.242 m/s), significantly different (*p* = 0.01) from the tight forward, loose forward, and half back positions. Tight forwards recorded the lowest maximum velocity (6,066 ± 1,079 m/s) and were significantly lower (*p* = 0.01) than all secondary positional groups. Half backs recorded the highest match intensity (77.7 ± 11.6 m/min), significantly higher than tight forwards and outside backs (*p* = 0.01).

Half backs recorded the highest distance within velocity zone 3 (760.6 ± 88.7 m), significantly higher than all secondary positional groups (*p* = 0.00). Outside backs recorded the highest distance within velocity zones 4 (336.6 ± 37.8 m), significantly different (*p* = 0.01) from tight and loose forwards, and 5 (219.9 ± 41.9 m), significantly different from tight forwards, loose forwards, and half backs (*p* = 0.01).

## Discussion

The current study aimed to investigate the in-match running demands of South African university rugby players by using GPS during match play for primary and secondary positional groups. To the authors’ knowledge, no studies that assessed the in-match running demands of South African university rugby players have been conducted to date, which makes it difficult to draw comparisons.

The findings of the current study were similar to studies by McLellan et al. (2011) and Austin and Kelly (2014) (both elite-level rugby leagues), and Reardon et al. (2015) (elite rugby). All these studies reported that backline players achieved a greater total distance covered than the forwards during matches. Results differed only in the number of meters ran where younger players were participants. Venter et al. (2011) analyzed under-19 players, but their findings differed from the trend where front row forwards covered the most distance followed by the outside backs. Austin and Kelly (2014), however, found that players from both positional groups recorded higher total distance averages than those seen in similar studies. This could be attributed to the fact that their study involved elite rugby league players, who played with only 13 players on a regulation-sized field. The current study supported earlier findings by Austin et al. (2011) on Super 14 rugby players where backline players covered greater distances than forwards.

In the current study, the half backs recorded the highest total distance covered, with the tight forwards recording the least total distance in meters. This result was contrary to the findings of Quarrie et al. (2013), where outside backs achieved the greatest total distance (5,950 ± 755 m), followed by half backs (5,756 ± 915 m). This finding could be attributed to differences in sampling and grouping of players or the increased ball in playtime of the modern game. Austin et al. (2011) reported similar results to the findings of the current study. The study found that the inside backs achieved the greatest total distances; however, the study made use of four positional groups and combined the half backs and inside backs (Austin et al., 2011). The results for total distances covered vary to some degree between studies published in the literature. Discrepancies, such as sampling, competition level, age, and period of playing time in the case of Venter et al. (2011) could have influenced the results. Uncontrollable elements, such as the tactical planning of coaches and player fitness, would also affect the accuracy of results during match play. Teams could have further been influenced by tournament laws, such as the VC where players must overcompensate while other players in the team must sit out during the power play. Because of a lack of studies conducted on the student athlete population and the VC competition itself, strong inferences cannot be made that the law changes influenced the total distances covered. The results of the players also do not differ greatly from what has already been reported in the literature.

Limited literature is available on high-speed meters covered for primary positional groups. Players in the study by Reardon et al. (2015) covered more high-speed meters than the current study. These higher totals were possible because the participants were elite rugby players. Possible reasons for the large differences could be the inability of the tight forwards to reach the minimum speed to register high-speed meters and the number of contact events involving the tight forwards. Our findings were similar to those reported by Reardon et al. (2015) for secondary positional groups where outside backs recorded the highest and tight forwards the lowest high-speed meters. Jones et al. (2015), however, noted that the inside backs covered the most and the tight forwards covered the least distance for meters at high speed. Both studies indicate that they made use of professional players for their data collection, where player level, competition laws, and coaching tactics may affect the results of recorded data. Players exposed to high-speed meters during training may be able to cope with match loads better when experiencing constant exposure to high-speed meters. In the study by Reardon et al. (2015), the teams might have played a more running-oriented game that allowed the forwards to register more meters covered but still have the outside backs covering more meters at high speeds. Jones et al. (2015) possibly recorded teams where the tactic was to use the inside backs group to control the attacks through the middle of the field. Line breaks and higher numbers of phases could have contributed to the high number of meters by inside backs.

The maximum velocity of the primary positional groups was significantly different for the full match, whereas literature reported that the backs attained the highest scores. Our results conform to current literature (Duthie et al., 2006; Reardon et al., 2015; Tee and Coopoo, 2015) at different competition levels. The results of the current study followed a trend indicating some consistency in recorded results. Differences in results could be determined by playing level and game plans where teams play to their strengths. The maximum velocity of different playing positions of the current followed similar trends within published literature. Reardon et al. (2015) reported individual positions maximum velocities, where outside backs’ positions (wingers and fullbacks) registered maximum velocities of 8.34 and 7.99 m s^–1^, respectively. Inside backs registered 8.05 m s^–1^ (Reardon et al., 2015), which were lower than that of the outside backs, and it corresponds with the results of the current study. Owen et al. (2015) noted that forwards, because of their size and weight, were not physically able to move at high speeds, unlike the lighter smaller backline players. Forwards are also tactically used for their physicality and not necessarily speed. Another possible reason is the forwards’ involvement in set pieces and phase play, which is generally slow, while backs are often already moving when they receive the ball. Another notable point is the space within which both positional groups operate in. Backs are in more open playing field, while forwards are used physically in slower phase play.

The backs recorded a higher match intensity average for the duration of the match when compared to the forwards, similar to Cunniffe et al. (2009) and Reardon et al. (2015). These high averages may be attributed to the player level because the participants were professional players. Tee and Coopoo (2015), however, reported results that differed from other studies; their study revealed that there was no statistical difference between the forwards and backs. The difference between the two groups, population fitness and game plan, may have resulted in such close scores. The inside backs and loose forwards recorded the second and third highest averages in the current study. This might be a result of the physical demands of each position, where the loose forwards are involved in rucking, tackling, and defensive work regularly compared to the inside backs and outside backs who cover more distance in single bouts and at greater speeds, but less frequently. Limited research has been conducted on match intensity, especially regarding the secondary positional groups. A possible explanation might be the practicality of the information, where coaches and researchers might not see the use of the data because they could analyze the total distance recorded. Match intensity combined with match analysis statistics might be able to distinguish player work rate during matches. Coaches could analyze a player’s effectiveness on the field after seeing a high match intensity recording from a GPS unit. Similarly, match intensity may offer an indication of player intensity during training providing an indication of intensity rather than only volume as in the case with total distance.

Limited information on the number of accelerations and decelerations in rugby has been published. The current study recorded higher acceleration and deceleration counts compared to previous research by Owen et al. (2015). However, only one-half of match play was recorded in the Owen et al. study to increase the sample size. This large difference in the number of accelerations and decelerations from the current study could be attributed to the inclusion and exclusion criteria used. Delaney et al. (2018) noted that the average velocity of most team sports was between 1.3 and 2.3 m/s (low intensity), which could question the ability of the players to accelerate and decelerate. Rugby as a stop-start, high-impact sport requiring players to accelerate and decelerate numerous times within matches was highlighted by the study on Super Rugby players by Owen et al. (2015).

The constant effort to move contributes to fatigue during play, affecting the ability to perform (Hewit et al., 2011). Dalen et al. (2016) focused on accelerations and decelerations among soccer players and highlighted the physical strain of accelerating and decelerating on players. Metrics such as acceleration, deceleration, mass, and velocity provide player load (Dalen et al., 2016). It is unclear whether there are trends within published literature in the number of accelerations and decelerations. It is difficult to compare literature because researchers analyze different aspects of accelerating and decelerating, such as Hewit et al. (2011); Owen et al. (2015), and Delaney et al. (2018) who reported on the forcefulness of accelerating and decelerating. Assessing the number of accelerations and decelerations may indicate match demands on players for soft tissue injury management strategies or adapted training to cope with the demands of matches.

The backs and back secondary groups outperformed both the forwards and forward secondary groups in terms of distance covered in all three zones for the duration of the match; this agrees with researched literature. Reardon et al. (2015) reported that scrumhalves and fly halves covered less distance when compared to half backs of the current study. The experimental law changes in VC should be considered, where teams may play within these velocity zones to outpace opponents and benefit from the extra points on offer for scoring. Reardon et al. (2015) did, however, report on all meters covered >5.0 m/s, lower velocities than the current study. Cahill et al. (2013) reported that scrumhalves registered higher than the reported meters in the current study, which can be attributed to the groupings of positions because scrumhalves were identified as individual positions. Quarrie et al. (2013), however, reported values of non-significant or higher recorded metrics for fly half players when viewing individual position results; this could justify the selected positional groups of the current study. As mentioned by Cahill et al. (2013); Owen et al. (2015), and Tee et al. (2017), scrumhalf and fly half positions are constantly involved in set and phase play, often as the link between the forwards and backs providing context to the roles of the position.

The limitations of this study were GPS units that malfunctioned, reducing the number of valid data points, small sample size, and recording only 17 matches. The study has highlighted the following limitations: (1) small sample size due to GPS malfunction, players and teams not meeting the GPS or minimum time requirements, and only recording two teams over one competitive season; (2) lack of normative values for velocity thresholds; (3) lack of normative values for velocity thresholds for both primary and secondary positional groups, as well as the use of absolute thresholds over individualized thresholds; (4) tactical substitutions of players further reducing sample size; (5) no consistent data on valid inclusion times for player data; and (6) no contact data recorded, which may have impacted the results seen for some metrics.

### Practical Implications

The coaching staff in a university rugby environment can identify player demands during match play and focus efforts on these areas during training. Table 4 represents the positional recommendations for primary and secondary positional groups based on match-recorded data. Season and session planning may be taken into consideration, or for priority matches and examination periods. Recorded data on player running demands may also provide an indication of player ability during match play, which could be paired with training data. Similarly, the coaching staff may plan or cancel sessions based on player load data, where specific session running demands could increase player injury risk. Coaching sessions that may have yielded superior or inferior results than expected could lead to changes in the planning of player training sessions. This form of player monitoring would ideally enhance the management of player loads. The possibility of individualized player profiles or primary and secondary positional profiles can assist teams with regard to accuracy in which they prepare and execute training. Adjustments to velocity zones for the different positional groups can be done to accurately represent player ability during training. The recorded data reported here, paired with video analysis, could provide an even better indication of player movement and tactical impacts on playing positions for the future of rugby at a university level in South Africa.

**TABLE 4:** Practical applications and recommendations based on recorded match demands.

Position	Results	Practical application
Forwards	Covered less total distance, lower maximum velocity, lower match intensity, and completed less accelerations and decelerations.	Focus on increasing overall fitness to improve total distance and match intensity scores. Expose the groups to a series of high-intensity running protocols and construct games and drills that expose the forwards to extensive or prolonged running at a higher match intensity. Contact at higher match intensities could also be integrated into training to add more positional specificity of game demands to the conditioning of the position.
Backs	Covered more total distance, attained a higher maximum velocity, had a higher match intensity, and completed more accelerations and decelerations.	Focus on increasing overall fitness. Focus on ability to accelerate and decelerate safely and effectively from high velocity. Repeat exposure to high-intensity and high-speed running to avoid detraining effects. The implementation of repeat speed, maximal aerobic speed running, and high-intensity gameplay to further expose players that could result in overreaching on running demands.
Tight forwards	Covered the least total distance, attained the lowest maximum velocity, lowest match intensity, and lowest accelerations and decelerations.	Focus on increasing overall fitness. Focus on improving ability to perform repeated outs of high intensity to assist with increasing match intensity. Maximal aerobic speed running and drills ensuring players are overloaded adequately to meet running demands during training. Variations in work-to-rest ratios and metabolic training such as tempo or lactate running could be used.
Loose forwards	Highest number of accelerations and decelerations. High total distance, maximum velocity, and match intensity.	Focus on overall fitness. Implement acceleration and deceleration strategies to expose players to high braking and acceleration loads. Repeat speed training to further expose players to forces of acceleration and deceleration to stimulate adaptations assisting the management of braking and accelerating forces on the body.
Half backs	Highest total distance Highest match intensity	Focus on overall running fitness. Implement work-to-rest ratios within training to maximize running efficiency. Expose players to high training intensities to simulate match conditions through repeat speed, maximal aerobic speed, and overspeed running.
Inside backs	High match intensity, maximum velocity, and total distance	Focus on overall fitness. Focus on maintaining high running velocity over longer distances. Repeated bouts of high intensity running or repeat speed, while adjusting the work to rest ratios stimulating adaptations for consistent high-speed running.
Outside backs	Highest maximum velocity	Focus on overall fitness. Focus on high speed running to expose players to longer periods of high-intensity running. Maximal speed running and repeat speed ability through varied rest periods during training can be implemented. The focus may be aimed at the quality of the speed meters covered rather than the distance.

## Conclusion

The major findings of the study note that backs covered higher speed meters and achieved higher maximum velocities than forwards for primary positional groups. The halfbacks covered the highest total distance and the most distance within velocity zone 3, and the outside back covered the highest high-speed meters among all secondary positional groups. Although the VC competition had a variety of law changes, many of the results remained significantly unchanged as seen in previous research listed in the “Discussion” section. As mentioned before, the involvement of the half back positions during a match may indicate the importance of conditioning in those specific positions for players to be able to handle the specific match loads. It is also important to note that no impact data have been recorded, where such data might better describe the lower totals of the tight forwards group involved in the set phases of play. Recorded data of the current study might serve as a stepping stone to in-match running demands in the South African university rugby context and stimulate further research on other aspects of this unique population and a changing game. It must be noted that only two universities were part of the study, and the results do not represent the whole university rugby population. Further research should be aimed at developing training programs catering to the demands of match play during training. These programs should be tailored to a specific metric, such as total distance or high-speed meters, and the implications thereof. Further research on player wellness during training demands spikes or increased loads to simulate match conditions, which leads to another avenue to explore, namely, player management.

## Ethics Statement

The studies involving human participants were reviewed and approved by Research Ethics Committee (HSREC) (UFS-HSD2017/0062) at the University of the Free State approved the study. The patients/participants provided their written informed consent to participate in this study.

## Data Availability Statement

The raw data supporting the conclusions of this article will be made available by the authors, without undue reservation.

## Author Contributions

CD collected the data and drafted the manuscript. All authors contributed to the article and approved the submitted version.

## Conflict of Interest

The authors declare that the research was conducted in the absence of any commercial or financial relationships that could be construed as a potential conflict of interest.
